# ﻿ *Sinoseneciominshanicus* (Asteraceae, Senecioneae), a new species from south-eastern Gansu and northern Sichuan, China

**DOI:** 10.3897/phytokeys.218.97475

**Published:** 2023-01-10

**Authors:** Xiu-Jiang Su, Wen-Qun Fei, Ding Zhao, Ying Liu, Qin-Er Yang

**Affiliations:** 1 Key Laboratory of Plant Resources Conservation and Sustainable Utilization, South China Botanical Garden, Chinese Academy of Sciences, Guangzhou 510655, Guangdong, China; 2 Administration Bureau of Baiyunshan Nature Reserve, Baojing 416500, Hunan, China; 3 University of Chinese Academy of Sciences, Beijing 100049, China; 4 Administration Bureau of Xuebaoding National Nature Reserve, Pingwu 622550, Sichuan, China; 5 State Key Laboratory of Biocontrol and Guangdong Key Laboratory of Plant Resources, School of Life Sciences, Sun Yat-sen University, No. 135, Xin-Gang-Xi Road, Guangzhou 510275, Guangdong, China; 6 Center of Conservation Biology, Core Botanical Gardens, South China Botanical Garden, Chinese Academy of Sciences, Guangzhou 510655, Guangdong, China

**Keywords:** chromosome number, Compositae, floral micromorphology, *Sinoseneciorotundifolius*, taxonomy

## Abstract

*Sinoseneciominshanicus* (Asteraceae, Senecioneae), a new species from south-eastern Gansu (Wenxian and Zhugqu counties) and northern Sichuan (Pingwu county), China, is described and illustrated. This species is similar to *S.rotundifolius*, a species locally endemic to Songpan county in northern Sichuan, in having a scapigerous habit, orbicular leaves and solitary capitula, but differs by the presence (vs. absence) of stolons and by having thinner rhizomes (ca. 2 mm vs. more than 5 mm in diameter), stems proximally sparsely fulvous arachnoid or glabrescent (vs. densely sericeous-villous) and obscure (vs. conspicuous) main veins on adaxial surface of leaves. The chromosome number of the new species is reported to be 2*n* = 60. Colour photographs of living plants in the wild and a distribution map are provided for the new species and *S.rotundifolius*. The geographical distribution of *S.rotundifolius* is also corrected, with the previous record of this species from south-eastern Gansu (Wenxian county) actually referring to *S.minshanicus*.

## ﻿Introduction

**Figure 1.**
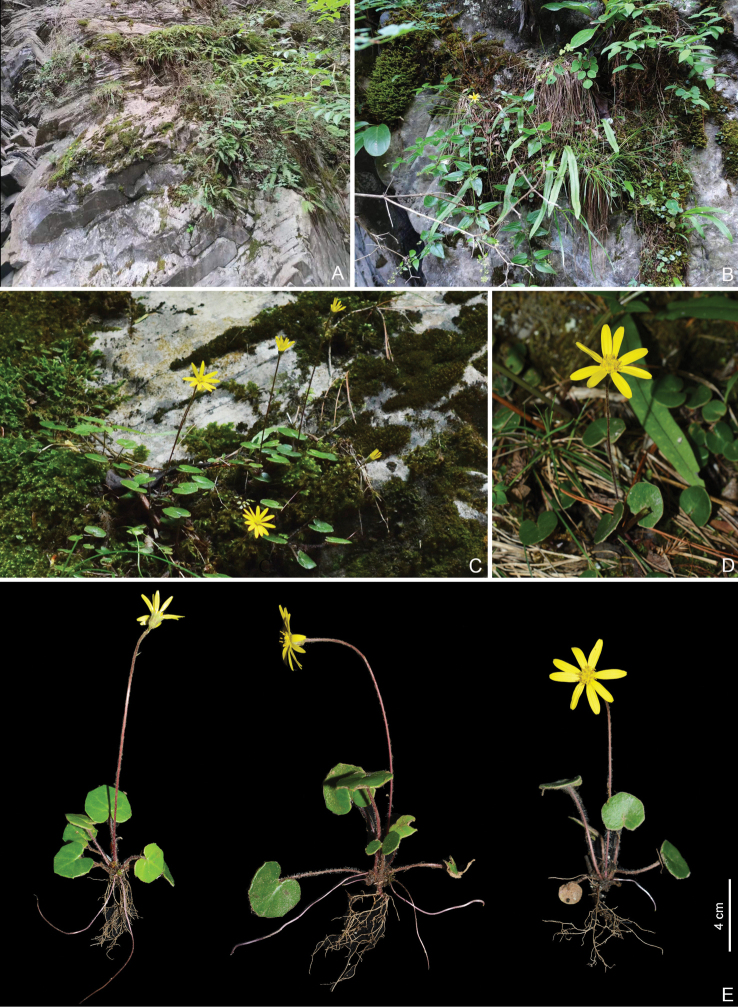
*Sinoseneciominshanicus* sp. nov. in the wild (China, Sichuan province, Pingwu county, the type locality) **A, B** habitat **C, D** habitat and habit **E** habit. Photographed by W.Q. Fei.

**Figure 2.**
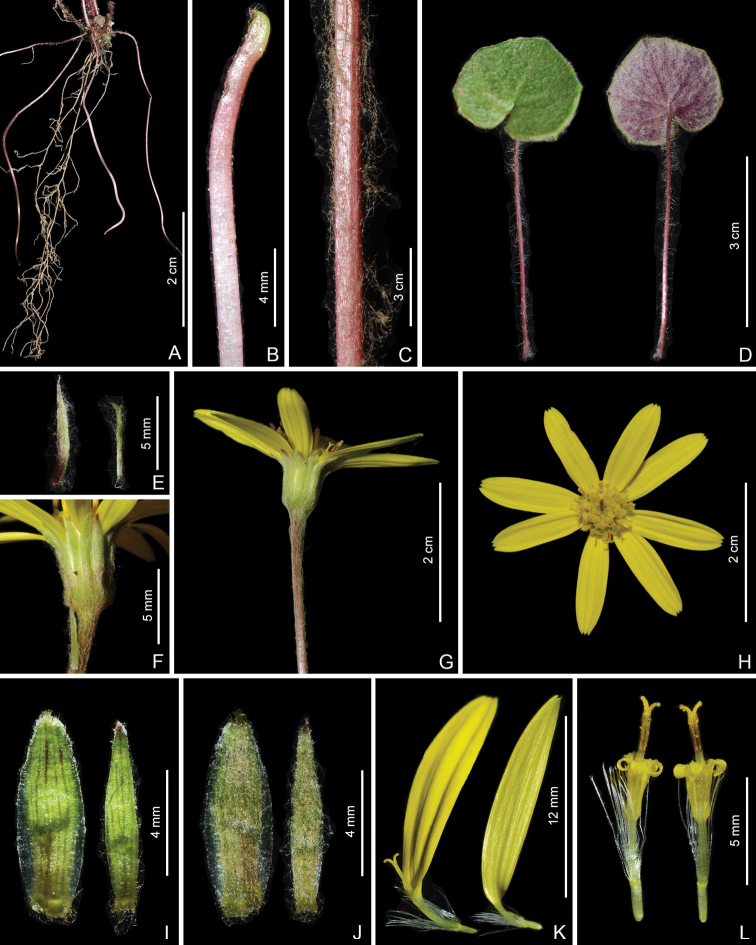
*Sinoseneciominshanicus* sp. nov. in the wild (China, Sichuan province, Pingwu county, the type locality) **A** stolons and roots **B** close-up of stolon **C** middle portion of scape **D** leaf (left: adaxial side; right: abaxial side) **E** bracts on the scape **F** close-up of capitulum (lateral view) **G** capitulum (lateral view) and distal portion of scape **H** capitulum (top view) **I** phyllaries (adaxial side) **J** phyllaries (abaxial side) **K** ray florets **L** disc florets. Photographed by W.Q. Fei.

**Figure 3.**
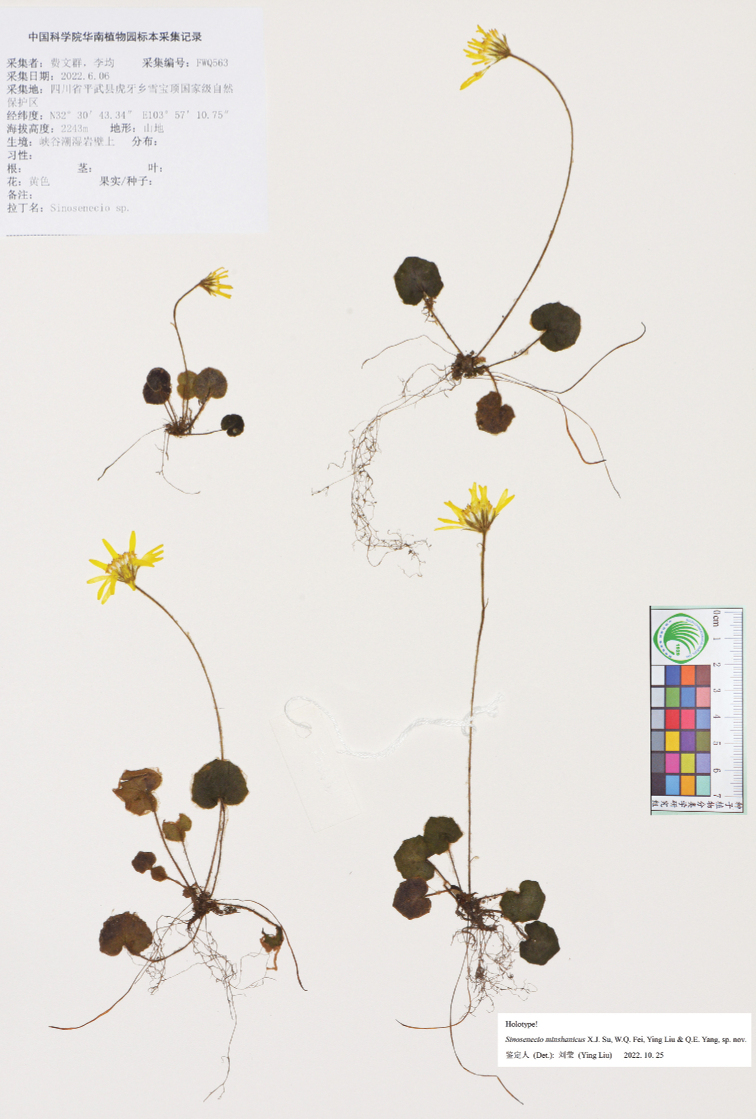
Holotype sheet of *Sinoseneciominshanicus* sp. nov.

**Figure 4.**
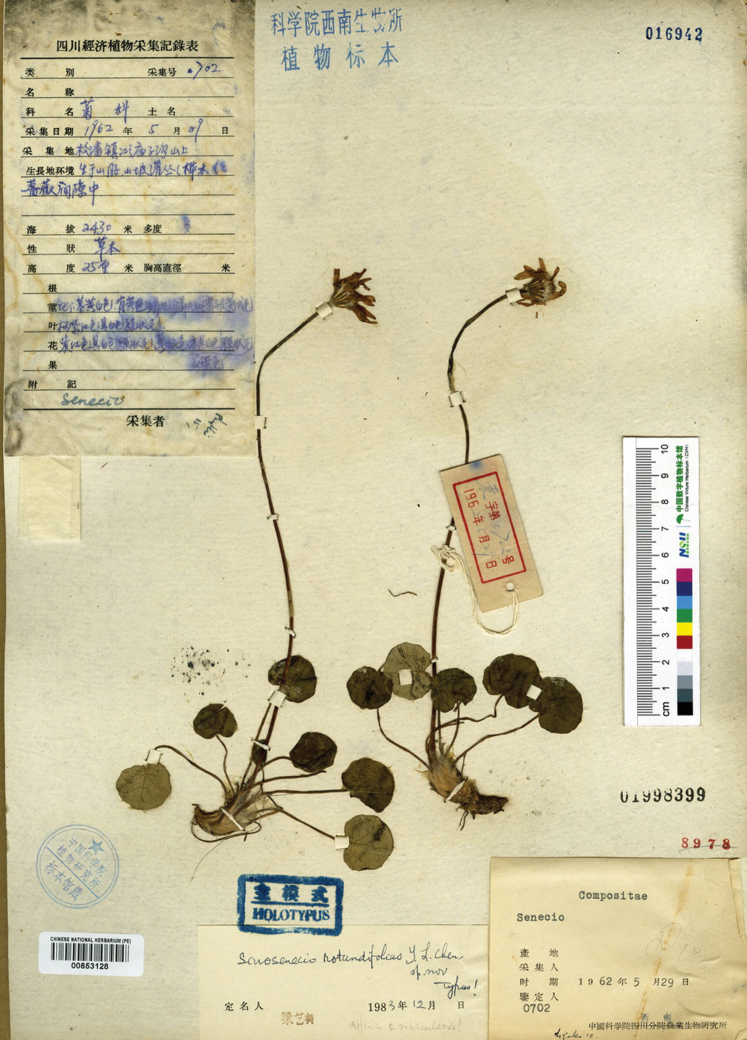
Holotype sheet of *Sinoseneciorotundifolius*.

**Figure 5.**
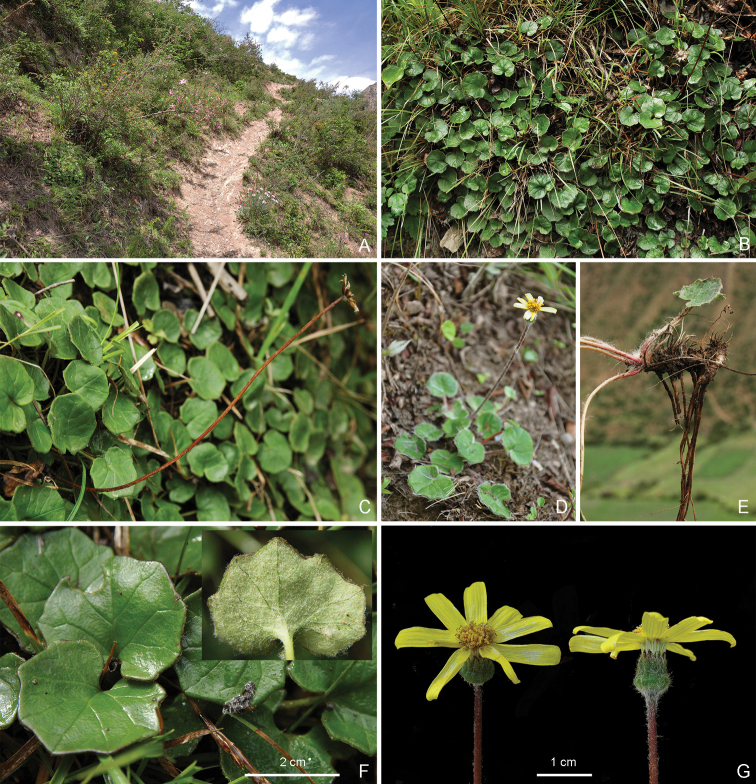
*Sinoseneciorotundifolius* in the wild (China, Sichuan province, Songpan county, the type locality) **A** habitat **B–D** habit **E** basal part of an individual with rhizome and roots, showing the densely sericeous-villous collar, stout rhizome and absence of stolons **F** leaves (adaxial side; inset: abaxial side) **G** capitula (left: top view; right: lateral view). Photographed by Ying Liu.

**Figure 6.**
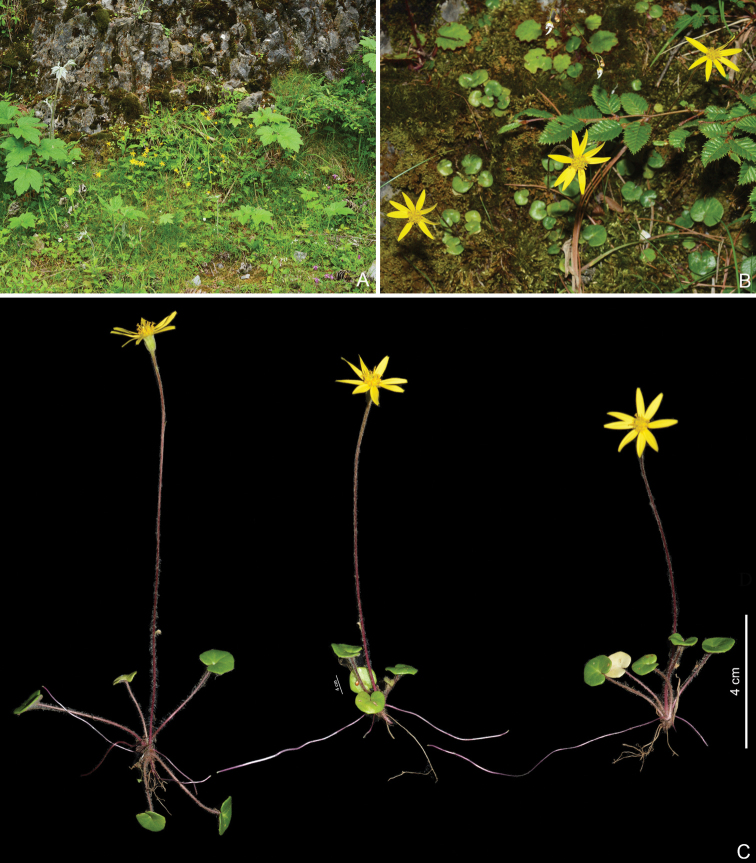
*Sinoseneciominshanicus* sp. nov. in the wild (China, Gansu province, Zhugqu county) **A** habitat **B** habitat and habit **C** habit. Photographed by W.Q. Fei.

**Figure 7.**
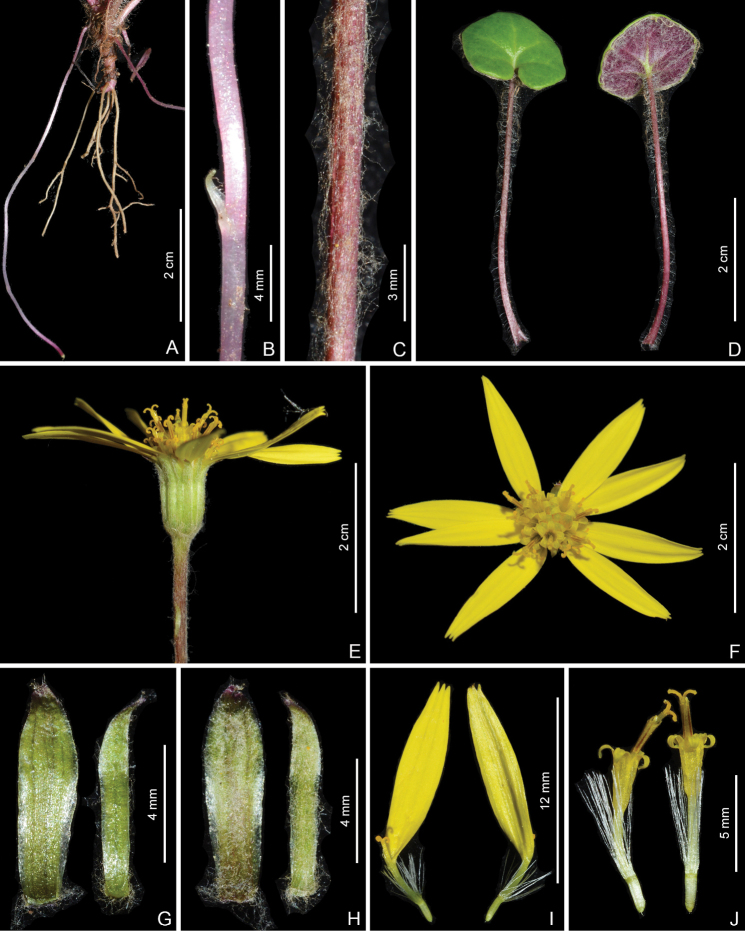
*Sinoseneciominshanicus* sp. nov. in the wild (China, Gansu province, Zhugqu county) **A** stolons and roots **B** close-up of stolon **C** middle portion of scape **D** leaf (left: adaxial side; right: abaxial side) **E** capitulum (lateral view) and distal portion of scape **F** capitulum (top view) **G** phyllaries (adaxial side) **H** phyllaries (abaxial side) **I** ray florets **J** disc florets. Photographed by W.Q. Fei.

During a botanical trip in 2016 in connection with the biodiversity survey of the Xuebaoding National Nature Reserve in Pingwu county in northern Sichuan province, China, we discovered an unusual population of *Sinosenecio* B. Nord. (Asteraceae, Senecioneae) (Figs 1–3). At first glance, the plants most closely resemble those of *S.rotundifolius* Y.L. Chen in having a scapigerous habit, orbicular leaves and solitary capitula, but are distinguishable immediately by having long and slender stolons (Figs 1E, 2A, B and 3). Closer examination revealed that the population in question is distinct from *S.rotundifolius* also by having thinner rhizomes (ca. 2 mm vs. more than 5 mm in diameter), stems proximally sparsely fulvous arachnoid or glabrescent (vs. densely sericeous-villous) and obscure (vs. conspicuous) main veins on adaxial surface of leaves (Figs 1E, 2D, 4, 5E and F). Moreover, the plants of the population prefer shaded and moist habitats on rocky cliffs and slopes along stream sides (Fig. 1A–D), while *S.rotundifolius* has been found only amongst *Betula* or *Rosa* bushes on arid slopes (Fig. 5A). During a botanical trip to Zhugqu county in south-eastern Gansu province in 2022, we discovered a population of *Sinosenecio* with the same morphological characters and habitats (Figs 6–7) as the population in northern Sichuan. We therefore determined that these two populations represent a hitherto undescribed species. Our re-examination of two collections identified on the determination slips or cited as *S.rotundifolius* by Liu (2010), *Baishuijiang Exped. 0320* (PE; http://www.cvh.ac.cn/spms/detail.php?id=eaffdad4) and *Baishuijiang Exped. 0800* (PE; http://www.cvh.ac.cn/spms/detail.php?id=eaffdb62), from Wenxian county in south-eastern Gansu revealed that they also belong to this new species. This species is described below and its somatic chromosome number (2*n*) is also reported.

## ﻿Taxonomic treatment

### ﻿ Sinosenecio minshanicus

Taxon classificationPlantaeAsteralesAsteraceae

XiuJ.Su, W.Q.Fei, YingLiu & Q.E.Yang sp. nov.

F39CB994-AA17-55A9-879E-078FE31949B7

urn:lsid:ipni.org:names:77311682-1

Figs 1 , 2 , 3 , 6 and 7

#### Type.

China. Sichuan province: Pingwu county, Huya town, Xuebaoding National Nature Reserve, on moist rocky cliff in valley, alt. ca. 2240 m, 6 June 2022, *W.Q. Fei & J. Li 563* (holotype: IBSC; isotypes: CDBI, PE, SYS). Fig. 3.

#### Diagnosis.

*Sinoseneciominshanicus* most closely resembles *S.rotundifolius* in having a scapigerous habit, orbicular leaves and solitary capitula, but differs by the presence (vs. absence) of stolons and by having thinner rhizomes (ca. 2 mm vs. more than 5 mm in diameter), stems proximally sparsely fulvous arachnoid or glabrescent (vs. densely sericeous-villous) and obscure (vs. conspicuous) main veins on adaxial surface of leaves.

#### Description.

**Figure 8.**
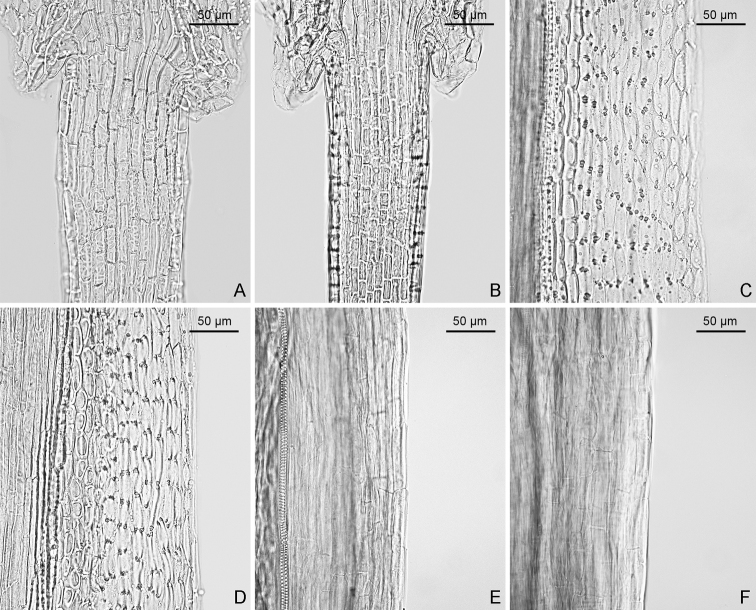
Two floral micromorphological characters (**A–D**) and achene surface (**E, F**) of two populations of *Sinoseneciominshanicus* sp. nov. **A, B** uniformly-sized cells of filament collar of stamens **C, D** strictly polar endothecial cell wall thickenings **E, F** smooth achene surface. Voucher: **A, C, E** from *W.Q. Fei & J. Li 598* (IBSC, SYS) from Zhugqu county in south-eastern Gansu, China **B, D, F** from *W.Q. Fei & J. Li 563* (CDBI, IBSC, PE, SYS) from Pingwu county in northern Sichuan province, China.

**Figure 9.**
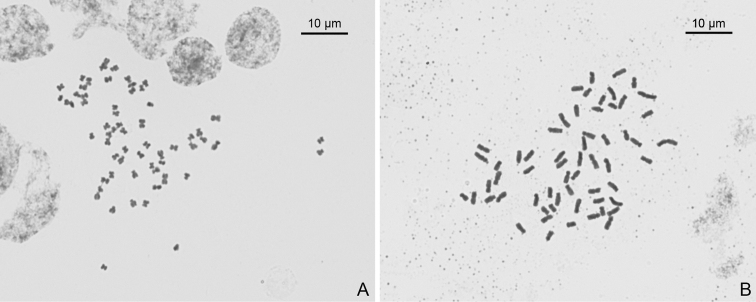
Mitotic metaphase chromosomes (2*n* = 60) of two populations of *Sinoseneciominshanicus* sp. nov. **A** population from Zhugqu county in south-eastern Gansu province, China; voucher: *W. Q. Fei & J. Li 598* (IBSC, SYS) **B** population from Pingwu county in northern Sichuan province, China; voucher: *W.Q. Fei & J. Li 563* (CDBI, IBSC, PE, SYS).

Scapigerous herbs with axillary slender stolons. Rhizomes short, ca. 2 mm in diameter, with few fibrous roots. Stems solitary, erect, purplish, scapiform, 7.5–17 cm tall, simple, proximally sparsely fulvous arachnoid or glabrescent, distally fulvous pubescent with uniseriate hairs or glabrescent. Leaves radical, rosulate; petioles 0.5–5 cm long, slender, basally expanded, pubescent with uniseriate hairs; blades orbicular or reniform-orbicular, 0.5–1.7 × 0.7–2.3 cm, subleathery, abaxially purplish, fulvous arachnoid, adaxially green, glabrous, palmately 5-veined, veins obscure adaxially and slightly raised abaxially, margin subentire or repand and mucronulate, base cordate, apex acute or rounded. Capitula terminal, solitary, radiating, 2.5–3.4 cm in diameter, scape with 1–3 linear bracts 2–5 mm long in middle or upper parts. Involucres long-campanulate, ca. 5 × 7–10 mm, ecalyculate, fulvous pubescent; phyllaries 10–14, oblong-lanceolate, 1–2 mm wide, margin narrowly scarious, apically purplish, fulvous pubescent. Ray florets 8–12; tube 3–3.5 mm long; lamina yellow, oblong, 10–14 × 2–3 mm, 4-veined, apically denticulate. Disc florets 15–32; corolla yellow, ca. 6 mm long, with ca. 2.5 mm long tube and funnel-form campanulate limb; lobes ovate-lanceolate, apically acuminate. Anthers oblong, 2.5 mm long, basally obtuse to rounded, appendages ovate-lanceolate; anther-collar cells uniform (Fig. 8A and B); endothecial cell wall thickenings strictly polar (Fig. 8C and D). Style branches 0.7 mm long, recurved, apically truncate, papillose. Achenes (immature) cylindrical, ca. 2 mm long, glabrous, smooth, ribbed (Fig. 8E and F). Pappus white, 4–6 mm long. 2*n* = 60 (Fig. 9A and B).

#### Phenology.

Flowering in June; fruiting in July.

#### Etymology.

The specific epithet, “*minshanicus*”, is derived from Min Shan, a chain of mountains extending from south-western Gansu to northern Sichuan, China. The currently known localities of the new species are all situated in the Minshan Mountains region.

#### Distribution.

**Figure 10.**
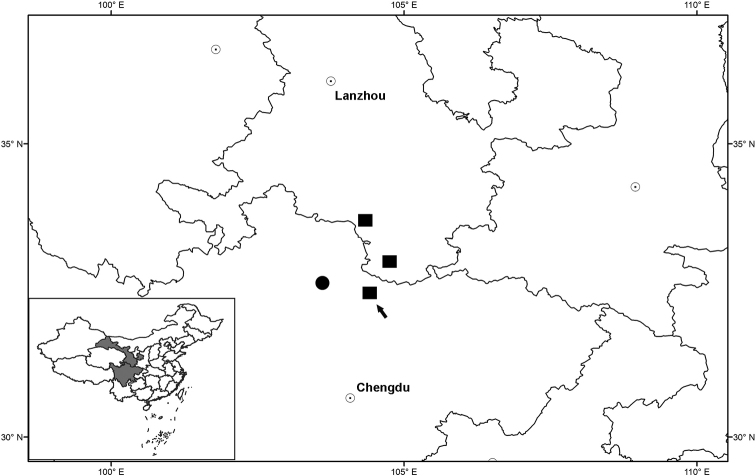
Distribution of *Sinoseneciominshanicus* sp. nov. (■) and *S.rotundifolius* (●). The arrow indicates the type locality, i.e. Pingwu county in Sichuan province, China.

*Sinoseneciominshanicus* is currently known from south-eastern Gansu (Wenxian and Zhugqu counties) and northern Sichuan (Pingwu county), China (Fig. 10). It grows on shaded and moist places in forests or on rocky cliffs and slopes along stream sides at altitudes of 2200–3000 m above sea level.

#### Additional specimens examined.

China. Gansu province: Wenxian county, Baishuijiang Nature Reserve, Qiujiaba, on slope in forest, alt. 2500 m, 26 June 2006, *Baishuijiang Exped. 0320* (PE); Wenxian county, Baishuijiang Nature Reserve, in *Abies* and *Rhododendron* forests, alt. 3000 m, 30 June 2006, *Baishuijiang Exped. 0800* (PE); Zhugqu county, Chagang forestry station, in grasses and mosses on shaded rocky slopes, alt. ca. 2400 m, 21 June 2022, *W. Q. Fei & J. Li 598* (IBSC, SYS).

#### Conservation status.

*Sinoseneciominshanicus* is currently known from Wenxian and Zhugqu counties in south-eastern Gansu and Pingwu county in northern Sichuan, China. Only approximately 30 to 50 mature individuals were discovered each in the Pingwu and Zhugqu populations. They are scattered within ca. 1 km along a valley. Data of the size of the two Wenxian populations are not available. Although the known populations of *S.minshanicus* are all located within national nature reserves, some human activities, road building in particular, may destroy their habitats and, thus, severely affect the survival of this species. According to the IUCN Red List Categories and Criteria (IUCN 2012), *S.minshanicus* may better be categorised as Endangered (EN).

#### Notes.

The genus *Sinosenecio* as defined by Chen et al. (2011) encompasses two major groups of species, with one having strictly polar anther endothecial cell wall thickenings and base chromosome number of *x* = 30 and occurring in mountainous areas largely surrounding the Sichuan basin in south-western China and the other having polar and radial thickenings and base chromosome number of *x* = 24 (rarely 13) and largely occurring in mountainous areas in central and southern China (Liu 2010; Chen et al. 2011; Liu and Yang 2011a, b, 2012; Liu et al. 2019; Zou et al. 2020; Chen et al. 2022; Peng et al. 2022; Su et al. 2023). Obviously, *S.minshanicus* belongs to the first group, in which 15 species are currently recognised, including *S.homogyniphyllus* (Cumm.) B. Nord., the type species of *Sinosenecio* and *S.rotundifolius*, the putative closest ally of *S.minshanicus* (Liu 2010; Chen et al. 2011; Chen et al. 2022; Su et al. 2023). In this group, *S.minshanicus* is the only species hitherto known to have slender stolons.

*Sinoseneciorotundifolius* was described on the basis of a single collection, *Inst. Biol. Sichuan Exped. 702* (Fig. 4), from Songpan county in northern Sichuan, China (Chen 1988). It was recorded to be locally endemic to Songpan in the account of *Sinosenecio* in the “Flora Reipublicae Popularis Sinicae” (Chen 1999). The results of our observation of living plants of this species from the type locality are shown in Fig. 5. As mentioned earlier, Liu (2010) cited two collections, *Baishuijiang Exped. 0320* (PE) and *Baishuijiang Exped. 0800* (PE), from Wenxian county in south-eastern Gansu under *S.rotundifolius*, thus extending the distributional range of this species. This treatment was adopted by Chen et al. (2011) in the account of *Sinosenecio* in the “Flora of China”. We re-examined these two collections and found that they have slender stolons and match *S.minshanicus* well also in other characters and thus belong to this species. The previous record of *S.rotundifolius* from south-eastern Gansu actually refers to *S.minshanicus*. Currently *S.rotundifolius* is indeed known only from its type locality in Songpan county in northern Sichuan, not occurring in south-eastern Gansu. As pointed out by Jeffrey and Chen (1984), *Sinosenecio* is noteworthy for the narrow endemism of many of its species.

*Sinoseneciominshanicus* occurs in the same valley together with another species of the same group in the genus, i.e. *S.pingwuensis* Xiu J. Su et al. (Su et al. 2023). Both of them prefer shaded and moist microhabitat and share the same flowering time (June). However, we did not observe any morphologically putative hybrids between them, which is probably due to isolation via intrinsic post-zygotic barriers.

## Supplementary Material

XML Treatment for Sinosenecio minshanicus

## References

[B1] Chen YL (1988) Eight new species of the tribe Senecioneae (Compositae) from China. Zhiwu Fenlei Xuebao 26(1): 50–57.

[B2] Chen YL (1999) Compositae-Senecioneae. In: Chen YL (Ed.) Flora Reipublicae Popularis Sinicae Vol.77 (1). Science Press, Beijing, 1–358.

[B3] Chen YL Liu Y Yang QE Nordenstam B Jeffrey C (2011) *Sinosenecio* B. Nordenstam. In: Wu ZY Raven PH Hong DY (Eds) Flora of China Vol.20–21. Science Press, Beijing, 464–481.

[B4] Chen B Liu Y Luo JX Wang Q Yang QE (2022) *Sinoseneciojiuzhaigouensis* (Asteraceae, Senecioneae), a new species from Sichuan, China. Phytotaxa 544(3): 289–294. 10.11646/phytotaxa.544.3.3

[B5] IUCN (2012) IUCN Red List Categories and Criteria: Version 3.1. 2^nd^ edn. Gland, Switzerland and Cambridge, UK, 1–32.

[B6] Jeffrey C Chen YL (1984) Taxonomic studies on the tribe Senecioneae (Compositae) of Eastern Asia. Kew Bulletin 39(2): 205–446. 10.2307/4110124

[B7] Liu Y (2010) Systematics of the genus *Sinosenecio* B. Nord. (Asteraceae). Ph.D. thesis, Institute of Botany, Chinese Academy of Sciences, Beijing, 1–277.

[B8] Liu Y Yang QE (2011a) Cytology and its systematic implications in *Sinosenecio* (Senecioneae-Asteraceae) and two closely related genera. Plant Systematics and Evolution 291(1–2): 7–24. 10.1007/s00606-010-0365-3

[B9] Liu Y Yang QE (2011b) Floral micromorphology and its systematic implications in the genus *Sinosenecio* (Senecioneae-Asteraceae). Plant Systematics and Evolution 291(3–4): 243–256. 10.1007/s00606-010-0385-z

[B10] Liu Y Yang QE (2012) *Sinoseneciojiangxiensis* (Asteraceae), a new species from Jiangxi, China. Botanical Studies (Taipei, Taiwan) 53(3): 401–414.

[B11] Liu Y Xu Y Yang QE (2019) *Sinoseneciopeltatus* (Asteraceae, Senecioneae), a remarkably distinctive new species from Guangdong, China. Phytotaxa 406(3): 206–212. 10.11646/phytotaxa.406.3.7

[B12] Peng JY Zhang DG Deng T Huang XH Chen JT Meng Y Wang Y Zhou Q (2022) *Sinosenecioyangii* (Asteraceae), a new species from Guizhou, China. PhytoKeys 210(34): 1–13. 10.3897/phytokeys.5555.89480 PMC9848965 36760415

[B13] Su XJ Fei WQ Liu Y Yang QE (2023) (in press) *Sinoseneciopingwuensis* (Asteraceae, Senecioneae), a new species from northern Sichuan, China. PhytoKeys. 10.3897/phytokeys.218.97485 PMC9853224 36762275

[B14] Zou CY Liu Y Liu Y (2020) *Sinosenecioovatifolius* (Asteraceae), a new species from Guangxi, China. Phytotaxa 460(2): 149–159. 10.11646/phytotaxa.460.2.5

